# Multimodal Simon Effect: A Multimodal Extension of the Diffusion Model for Conflict Tasks

**DOI:** 10.3389/fnhum.2018.00507

**Published:** 2019-01-09

**Authors:** Mohammad-Ali Nikouei Mahani, Karin Maria Bausenhart, Majid Nili Ahmadabadi, Rolf Ulrich

**Affiliations:** ^1^Cognition and Perception, Department of Psychology, University of Tübingen, Tübingen, Germany; ^2^Cognitive Systems Lab, School of Electrical and Computer Engineering, College of Engineering, University of Tehran, Tehran, Iran

**Keywords:** conflict processing, simon task, multimodal congruency effect, diffusion model for conflict tasks (DMC), reaction time, multisensory processing

## Abstract

In conflict tasks, like the Simon task, it is usually investigated how task-irrelevant information affects the processing of task-relevant information. In the present experiments, we extended the Simon task to a multimodal setup, in which task-irrelevant information emerged from two sensory modalities. Specifically, in Experiment 1, participants responded to the identity of letters presented at a left, right, or central position with a left- or right-hand response. Additional tactile stimulation occurred on a left, right, or central position on the horizontal body plane. Response congruency of the visual and tactile stimulation was orthogonally varied. In Experiment 2, the tactile stimulation was replaced by auditory stimulation. In both experiments, the visual task-irrelevant information produced congruency effects such that responses were slower and less accurate in incongruent than incongruent conditions. Furthermore, in Experiment 1, such congruency effects, albeit smaller, were also observed for the tactile task-irrelevant stimulation. In Experiment 2, the auditory task-irrelevant stimulation produced the smallest effects. Specifically, the longest reaction times emerged in the neutral condition, while incongruent and congruent conditions differed only numerically. This suggests that in the co-presence of multiple task-irrelevant information sources, location processing is more strongly determined by visual and tactile spatial information than by auditory spatial information. An extended version of the Diffusion Model for Conflict Tasks (DMC) was fitted to the results of both experiments. This Multimodal Diffusion Model for Conflict Tasks (MDMC), and a model variant involving faster processing in the neutral visual condition (FN-MDMC), provided reasonable fits for the observed data. These model fits support the notion that multimodal task-irrelevant information superimposes across sensory modalities and automatically affects the controlled processing of task-relevant information.

## Introduction

People sometimes need to suppress task-irrelevant information to minimize interference with processing of task-relevant information. Standard examples for the empirical investigation of such situations are conflict tasks, such as the Stroop task, the Eriksen-Flanker task, and the Simon task (Stroop, 1935; Simon and Wolf, 1963; Eriksen and Eriksen, 1974). For instance, in the Simon task participants are instructed to respond to a non-spatial stimulus attribute (e.g., color, form, letter, or pitch) with a spatially defined response (e.g., a key press of the left or the right hand). Although the location of the stimulus presentation is task-irrelevant, it influences task performance. Specifically, responses are faster and more accurate when both the stimulus and the response are on the same spatial side (congruent condition) rather than on different sides (incongruent condition) (Simon and Wolf, 1963). Such congruency effects have been reported not only for the visual but also for other modalities (Simon and Rudell, 1967; Simon et al., 1970; Cohen and Martin, 1975; McClain, 1983; Jerger et al., 1988; Medina et al., 2014; Salzer et al., 2014). Thus, the effects of task-irrelevant information are not limited to a single modality but rather reveal a general phenomenon that presumably emerges from an amodal processing mechanism.

It has been suggested that this mechanism involves two separate processes acting simultaneously on stimulus input. More specifically, it is assumed that task-relevant information is processed by a controlled process, whereas task-irrelevant information is mediated by an automatic process. Moreover, these two processes are often assumed to operate in parallel and in independent pathways (Logan, 1980; Coles et al., 1985; Cohen et al., 1990; Hommel, 1993; Ridderinkhof, 2002). A recent quantitative account of this processing architecture is provided by an elaborated diffusion process model, called the Diffusion Model for Conflict Tasks (Ulrich et al., 2015). DMC is based on standard diffusion models according to which a decision-making process accumulates noisy task-relevant information until one of two decision boundaries is hit and the corresponding response is selected (Stone, 1960; Ratcliff, 1978; Ratcliff and Smith, 2004). This accumulation process is typically modeled as a standard Wiener diffusion process (Ratcliff, 1978). DMC extends this framework by adding a second process to incorporate processing of task-irrelevant information, which leads to a short-lived activation. This automatically triggered activation superimposes the activation from the controlled process, which operates on the task-relevant information. The superimposed activation from both processes triggers either the correct or the incorrect response. DMC can predict various phenomena associated with common conflict tasks, including reaction time (RT) patterns, the shape of conditional accuracy functions, and the shape of delta functions (Ulrich et al., 2015; Ellinghaus et al., 2018; White et al., 2018). Furthermore, DMC has been successfully linked to neurophysiological findings (Servant et al., 2016).

DMC's core assumption of independently operating controlled and automatic processes receives particular support from studies demonstrating that task-relevant and task-irrelevant information needs not to stem from the same stimulus source. Specifically, task-irrelevant information may impede performance even if it stems from a different modality than the task-relevant information. For example, task-irrelevant congruent auditory information decreases RT to visual stimuli compared to task-irrelevant incongruent auditory information (Simon and Craft, 1970; Donohue et al., 2013; Schupak et al., 2015). Similar cross-modal conflict effects have been reported for visual-tactile conflict tasks (Kennett et al., 2001; Spence et al., 2004; Yue et al., 2009; Wesslein et al., 2014; Poole et al., 2015). These results strengthen the notion that automatic and controlled processes act independently. Nevertheless, these results are limited to single sources of task-irrelevant information. Hence the question is, whether DMC accounts for the processing of multiple sources of task-irrelevant information as well.

The goal of the present study was thus to examine whether DMC can be extended to conflict tasks with two task-irrelevant information sources. Within DMC, it seems most reasonable that these conflicting sources are processed by two independent automatic processes, with each process generating separate activation and superimposing the controlled process. By orthogonally manipulating the compatibility of two independent task-irrelevant sources, it is possible to put this assumed superimposition within DMC to a comprehensive test.

To this end, we conducted two Simon task experiments with two rather than one task-irrelevant information sources (i.e., task-irrelevant location information was provided by two modalities). The first (second) experiment was a typical visual Simon task with additional task-irrelevant tactile (auditory) information. In both experiments, the spatial congruency of these task-irrelevant information sources (i.e., visual and tactile/auditory stimulus location) and the response side varied orthogonally. Specifically, stimulus location in both modalities could be congruent, neutral, or incongruent with the side of the correct response. DMC's architecture was extended to address the contribution of these two independent sources of automatic activation and the extended DMC was fitted to the experimental results of the two experiments. These experiments emulated the Simon task of the original DMC study (Ulrich et al., 2015) in order to facilitate the comparison of results between the present experiments and this former study.

## Experiment 1

This experiment examines whether task-irrelevant tactile stimulation influences speeded decisions in a visual Simon task. The spatial position of the tactile stimulation was congruent, incongruent, or neutral with the required response to a letter appearing to the left, to the right, or at the central position of the fixation point.

### Method

#### Participants

Thirty participants (26 women and 4 men) volunteered in this experiment (23.5 ± 3.5 years of age). They all reported normal or corrected-to-normal vision, normal tactile sense, and no neurological problems. All procedures and experimental protocols are approved by the ethical committee board of the University of Tehran and all methods were carried out in accordance with the approved guidelines. A written informed consent was also obtained from all participants prior to data collection and they either received 8 € per hour or course credit for their participation.

#### Stimuli and Apparatus

**Figure 1:**
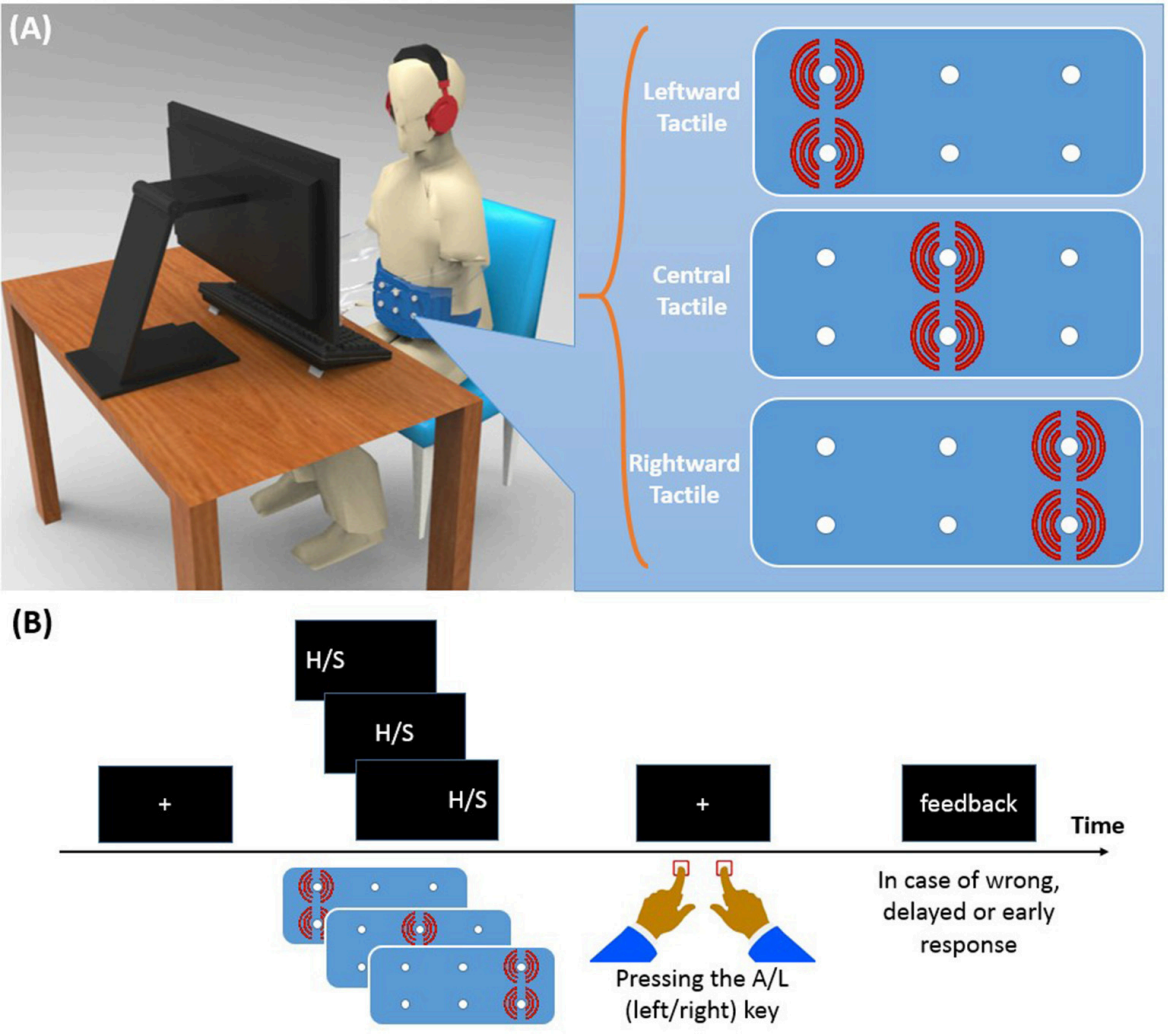
**(A)** Experimental setup and vibrotactile belt. Running two vibration motors on the left/center/right side of the belt causes a tactile stimulus to the left/center/right side of the participant's waist. **(B)** Time course of a trial. A left/center/right visual stimulus was presented along with a left/center/right tactile stimulus. Participants were asked to identify the visual stimulus (H or S) with a left/right key press and to ignore the location of the visual stimulus.

Participants were seated in a sound-attenuated room in front of a 19” CRT screen on which the visual stimuli were presented (see Figure 1A). The distance between participants' eyes and the monitor screen was about 50 cm. Like in Ulrich et al. (2015), we employed letters as imperative stimuli. Specifically, these were the letter “H” and “S” (Font: Arial; letter size: 48 pt, ~1.5° visual angle) which were presented in white color against a black background. One of these two letters appeared either to the left or to the right (8° visual angle) from of the center of the screen, or at the center. The tactile stimulus came from a custom canvas belt consisting of six vibration motors. Two motors were placed on the left, two at the center, and two on the right of the belt. Running vibration motors on the left/center/right side of the belt causes a tactile stimulus to the left/center/right side along the participant's horizontal body plane. Since participants varied in waist size, we designed two belts: a medium size belt and a large size belt. In the medium (large) size belt, the horizontal distance of motors was 16.5 (19.0) cm and the vertical distance was 1.3 (1.5) cm. The vibration motors were controlled by a XMEGA microcontroller with a 32 MHz clock, the same device as used in a previous study (Mahani et al., 2017). Stimulus durations of both visual and tactile stimuli were 200 ms. Oscilloscopic measurements were conducted to ensure simultaneity of the visual stimulation and the maximum vibration amplitude. Both the experiment and the microcontroller program were written in C++ language. Left and right responses were recorded with the “A” and “L” keys, respectively.

#### Procedure

Each trial of Experiment 1 started with the presentation of a fixation cross for 500 ms at the center of the screen (see Figure 1B). Then, the visual stimulus and the tactile stimulus were presented simultaneously for 200 ms. In both modalities, and independent of each other, stimuli were presented either on the left, on the right, or at the central position. For half of the participants, the stimulus “H” was associated with the left response key and “S” with the right response key. A reverse setting was used for the remaining participants. Participants were asked to ignore the location of the stimulation and to respond to the letter identity quickly within 1,500 ms, but also to avoid errors as much as possible. They received visual feedback for 1,000 ms when their RT was longer than 1,500 ms, or shorter than 150 ms, or if their response was wrong. The inter-trial delay was 1,000 ms.

#### Design

The combination of three visual positions (left, center, and right), three tactile positions (left, center, and right), and two letters (“H” and “S”) resulted in 18 trial types. Each trial type was repeated five times per block, and trials were presented in random order. Overall, participants completed six blocks. Note that the side of visual and tactile stimulation could be either congruent (same side), incongruent (opposite side), or neutral (central position) to the side of the correct response. Thus, from the orthogonal combination of the two within-subject factors Visual Congruency and Tactile Congruency, nine different conditions emerged: (1) Congruent visual and congruent tactile stimulation CVCT, (2) congruent visual and neutral tactile CVNT, (3) congruent visual and incongruent tactile CVIT, (4) neutral visual and congruent tactile NVCT, (5) neutral visual and neutral tactile NVNT, (6) neutral visual and incongruent tactile NVIT, (7) incongruent visual and congruent tactile IVCT, (8) incongruent visual and neutral tactile IVNT, and (9) incongruent visual and incongruent tactile IVIT. Each participant received each of the nine congruency conditions 60 times.

### Results

**Figure 2:**
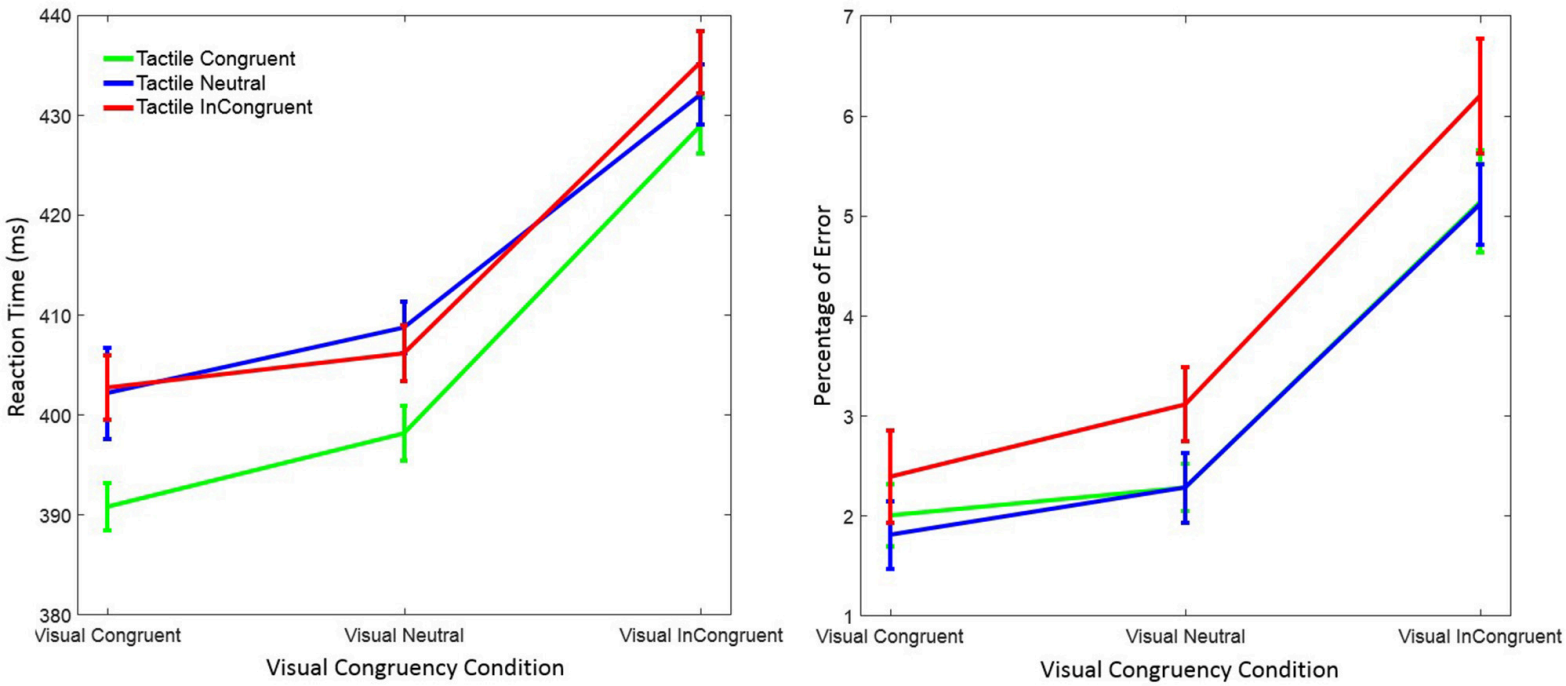
Mean reaction time (left figure) and mean percentage of response errors (right figure) in Experiment 1 as a function of visual and tactile congruency. Error bars were computed according to Morey's method (Morey, 2008).

Trials with RTs > 1,200 ms or < 150 ms were discarded (0.68%) from data analysis. However, statistical results were virtually identical when we kept those trials. Separate 3 x 3 within-subject ANOVAs with factors *visual congruency* (congruent, neutral, and incongruent) and *tactile congruency* (congruent, neutral, and incongruent) were performed on RT and on response errors. Figure 2 depicts mean RT and the percentage of response errors as a function of visual and tactile congruency.

#### Reaction Time

RT was significantly affected by visual congruency, *F*_(2, 58)_ = 62.74, *p* < 0.001, $\eta_{p}^{2}$ = 0.68, and tactile congruency, *F*_(2, 58)_ = 13.94, *p* < 0.001, $\eta_{p}^{2}$ = 0.33 ($\eta_{p}^{2}$ indicates the partial eta-squared). However, the interaction of the two factors was not significant, *F*_(4, 58)_ = 0.95, *p* = 0.43, $\eta_{p}^{2}$ = 0.03. *Post-hoc* Tukey tests showed that RTs were longer in the visual incongruent than in the visual neutral (*p* < 0.001), and in the visual congruent (*p* < 0.001) conditions. No significant difference was observed between the visual neutral and visual congruent condition (*p* = 0.20). *Post-hoc* Tukey tests for the tactile congruency conditions showed that RTs were shorter in the tactile congruent than in the tactile neutral (*p* < 0.001), and in the tactile incongruent (*p* < 0.001) conditions. The difference between the tactile incongruent and tactile neutral condition was not significant (*p* = 0.98).

#### Response Error

There were also significant main effects of visual congruency *F*_(2, 58)_ = 29.02, *p* < 0.001, $\eta_{p}^{2}$ = 0.50, and tactile congruency, *F*_(2, 58)_ = 7.93, *p* = 0.001, $\eta_{p}^{2}$ = 0.22, on mean response error. The interaction of visual and tactile congruency was not significant, *F*_(4, 58)_ = 0.39, *p* = 0.81, $\eta_{p}^{2}$ = 0.01. *Post-hoc* Tukey tests on visual congruency showed that the percentage of error was higher in the visual incongruent than in the visual neutral (*p* < 0.001), and in the visual congruent (*p* < 0.001) conditions. However, there was no difference between the visual neutral and the visual congruent condition (*p* = 0.36). The same Tukey test on tactile congruency revealed similar results; the percentage of errors was higher in the tactile incongruent than in the tactile neutral (*p* = 0.016), and in the tactile congruent (*p* = 0.002) conditions. The difference between the tactile congruent and tactile neutral conditions was not significant (*p* = 0.90).

#### Distributional Analysis of Reaction Time

**Figure 3:**
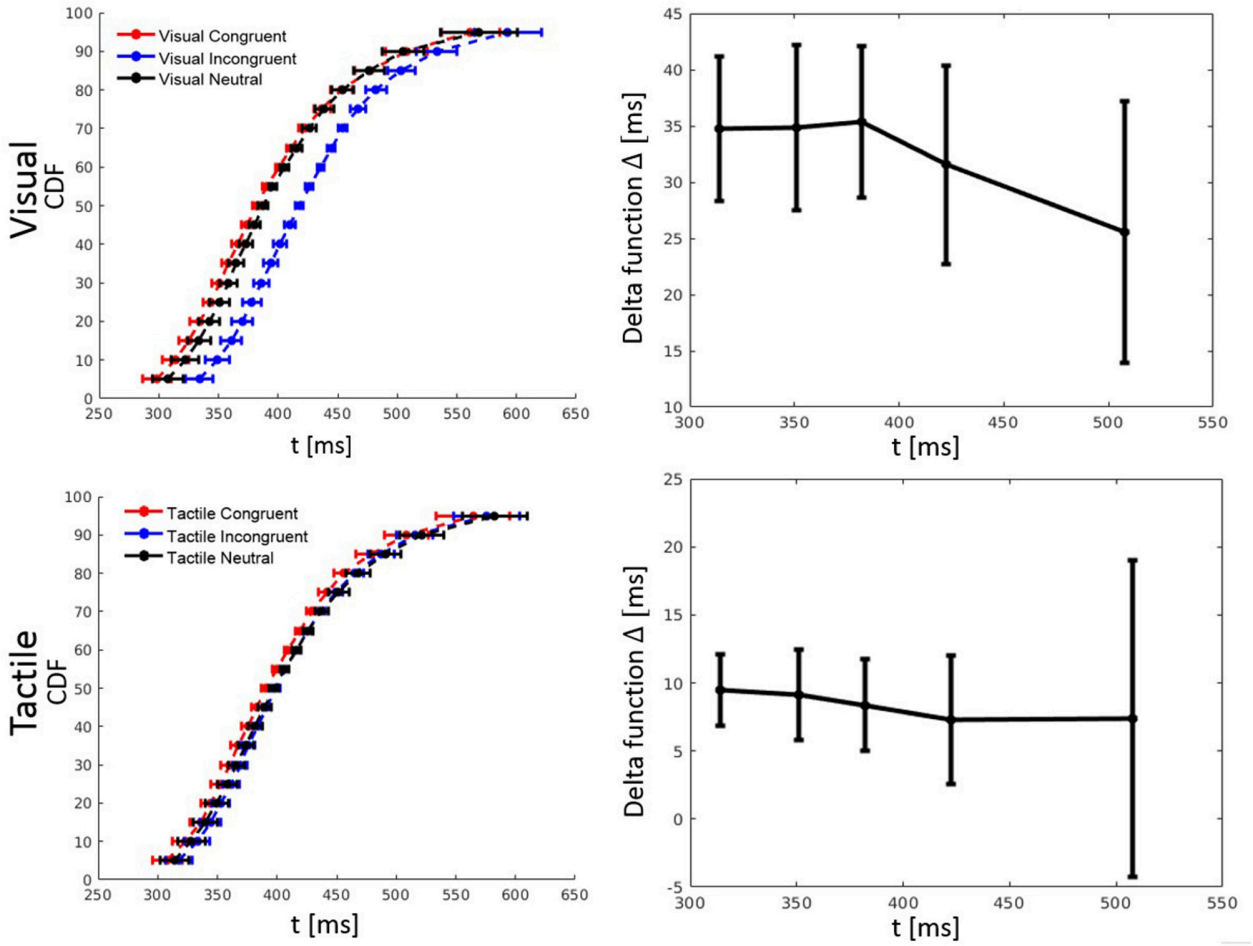
Cumulative distribution functions (CDFs) and delta (Δ) functions for percentiles (5, 10, 15, …, 95%) in Experiment 1. Error bars show 95% confidence intervals and are calculated according to Morey (2008). Each of the visual (tactile) CDFs was calculated as the average over all tactile (visual) congruency conditions. For example, the visual congruent CDF is the average of CVCT, CVNT, and CVIT conditions. Delta functions show the difference between the congruent and incongruent CDFs as a function of response time.

RT percentiles (10, 30, 50, 70, 90%) for each congruency condition and for each participant were estimated. Percentiles were analyzed by a three-way ANOVA with factors percentile, visual congruency, and tactile congruency. As one expects, the main effect of percentile was significant, *F*_(4, 116)_ = 228.74, *p* < 0.001, $\eta_{p}^{2}$ = 0.89. There was also a significant main effect of tactile congruency, *F*_(2, 58)_ = 12.82 *p* < 0.001, $\eta_{p}^{2}$ = 0.31, and visual congruency, *F*_(2, 58)_ = 64.91, *p* < 0.001, $\eta_{p}^{2}$ = 0.69. The three-way visual congruency x tactile congruency x percentile interaction was significant, *F*_(16, 464)_ = 1.76, *p* = 0.034, $\eta_{p}^{2}$ = 0.06. Figure 3 illustrates the cumulative distribution functions (CDFs) for all congruency conditions, as well as delta functions for the visual and tactile modalities. The CDF of each of the visual congruency conditions was averaged over all tactile congruency conditions (e.g., the visual congruent CDF is the average of the CVCT, CVNT, and CVIT conditions). The same approach was used to calculate CDFs for the tactile congruency conditions. Delta (Δ) functions show the percentile difference between the congruent and the incongruent condition for each modality.

#### Analysis of Conditional Accuracy Functions

Conditional accuracy functions (CAFs) depict response accuracy given response speed (**Figure 8**). As in previous investigations on conflict tasks, we have analyzed CAFs for each of the congruency conditions. All RTs of a given congruency condition were sorted from fastest to slowest. Thereafter, the RT distribution was split into five equal bins (0–20, 20–40, 40–60, 60–80, 80–100%) and the percentage of correct responses was calculated for each bin.

A three-way ANOVA with factors bin, visual congruency, and tactile congruency was used to analyze the CAFs. This analysis revealed a main effect of visual congruency, *F*_(2, 58)_ = 12.30, *p* < 0.001, $\eta_{p}^{2}$ = 0.30, and a main effect of tactile congruency, *F*_(2, 58)_ = 4.00, *p* = 0.024, $\eta_{p}^{2}$ = 0.12. However, the effect of bin on CAFs was not significant, *F*_(4, 116)_ = 1.88, *p* = 0.12, $\eta_{p}^{2}$ = 0.06. The three-way bin x visual congruency x tactile congruency interaction, *F*_(16, 464)_ = 0.75, *p* = 0.73, $\eta_{p}^{2}$ = 0.03, and all of the two-way interactions were not significant (all *F*s < 0.85 and all *p*s > 0.56).

### Discussion

We extended the Simon task to study the effect of simultaneous task-irrelevant tactile and task-irrelevant visual information on speeded visual decisions. The results show that both visual and tactile congruency significantly affected the task-relevant processing of letter identity. In general, visual and tactile incongruent stimulus locations produced longer RTs and more response errors than visual and tactile congruent stimulus locations, reflecting the typically expected pattern of results in the Simon task. In addition, *post-hoc* analyses showed that the effects regarding the neutral condition were not the same for the visual and tactile modalities. There was no significant difference between the visual congruent and the visual neutral condition in terms of both RT and response errors. Thus, only the visual incongruent information significantly increased RT and response errors. In contrast, there was no meaningful difference between the tactile incongruent and tactile neutral information in terms of RT while they had significantly different effects on the response errors. That is, for the tactile modality, the neutral condition was the same as the incongruent condition in terms of RT while it was the same as the congruent condition in terms of response errors.

## Experiment 2

This experiment assesses the effect of task-irrelevant auditory stimulation instead of task-irrelevant tactile stimulation on letter processing performance in the visual Simon task. Task-irrelevant tones were presented to the left or to the right ear, or to both ears simultaneously. Otherwise the experimental setup was identical to the one in Experiment 1. Thus, this experiment examines whether similar multimodal effects would emerge as in Experiment 1, when the task-irrelevant tactile information is replaced by task-irrelevant auditory information.

### Method

#### Participants

Thirty individuals (23.8 ± 3.0 years of age, 9 men and 21 women) participated in this experiment. They all reported normal or corrected-to-normal vision, and no neurological problems. All procedures and experimental protocols are approved by the ethical committee board of the University of Tehran and all methods were carried out in accordance with the approved guidelines. A written informed consent was also obtained from all participants prior to data collection. They either received 8 € per hour or course credit for their participation.

#### Apparatus and Procedure

In the second experiment, tactile stimuli were replaced by the auditory stimuli. The auditory stimuli came through Sony MDR-XD200 stereo headphones. The leftward (rightward) auditory stimulus was a mono sound provided to the left (right) ear and the central (neutral) stimulus was a stereo sound provided to both ears. The intensity of the mono and stereo stimuli were corrected using the binaural correction method (Epstein and Florentine, 2009) where the intensity of the mono stimulus was 75 dB (SPL) and the intensity of the stereo stimulus was 63 dB (SPL). The source of the auditory stimulus was a square wave with a frequency 440 Hz and a duration of 200 ms, that is, the same duration as the visual stimulus. The onsets of the visual and the auditory stimulus were synchronous. All other experimental details were identical to those of Experiment 1.

### Results

**Figure 4:**
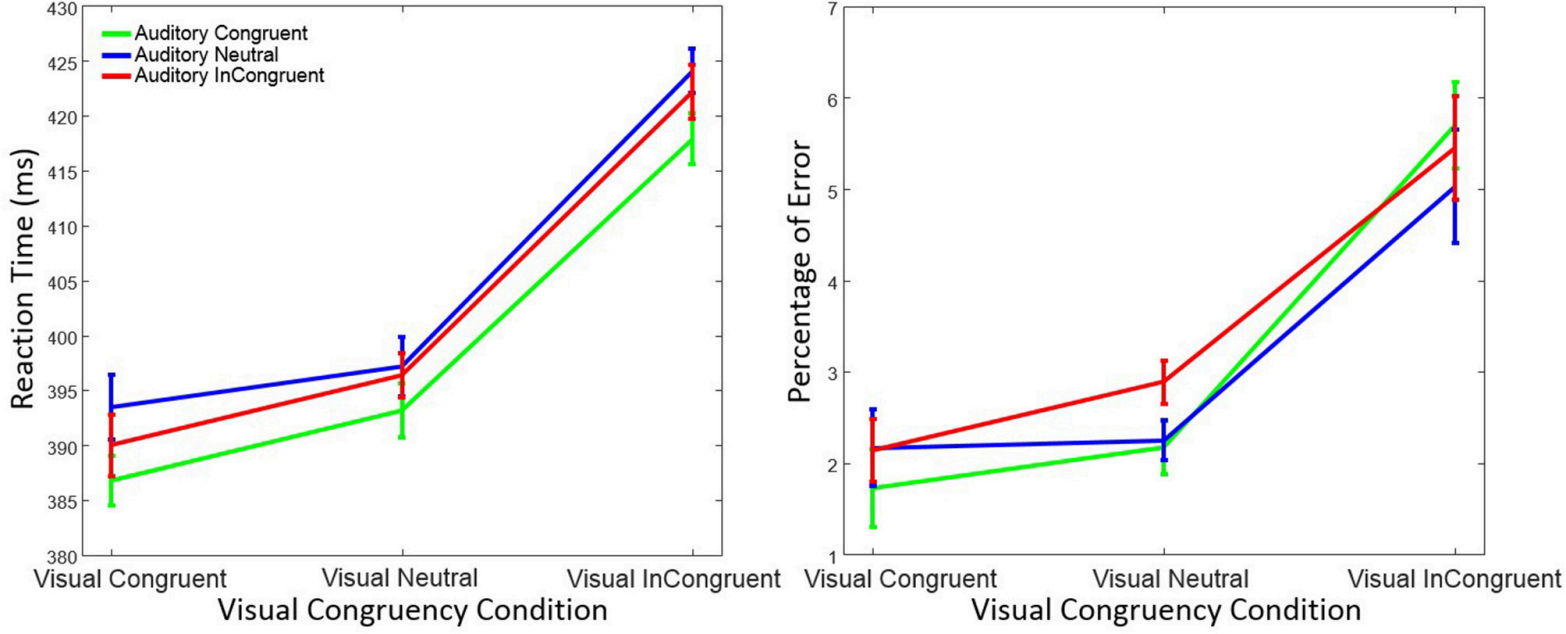
Mean reaction time (left figure) and mean percentage of response errors (right figure) in Experiment 2 as a function of visual and auditory congruency. Error bars were computed according to Morey's method (Morey, 2008).

As in Experiment 1, trials with RTs > 1,200 ms or < 150 ms were discarded (0.44%) from the data analysis. However, statistical results were virtually identical when these trials were included in the statistical analysis. Figure 4 shows mean RT and percentage of response errors as a function of visual and auditory congruency.

#### Reaction Time

RTs were again analyzed using a within-subject ANOVA with factors *visual congruency* (congruent, neutral, and incongruent) and *auditory congruency* (congruent, neutral, and incongruent). As before, there was a significant main effect on RT of visual congruency, *F*_(2, 58)_ = 80.76, *p* < 0.001, $\eta_{p}^{2}$ = 0.74, as well as of auditory congruency, *F*_(2, 58)_ = 4.19, *p* = 0.019, $\eta_{p}^{2}$ = 0.13. The interaction of visual x auditory congruency on RT was not significant, *F*_(4, 58)_ = 0.25, *p* = 0.90, $\eta_{p}^{2}$ = 0.01. A *post-hoc* Tukey test on auditory congruency illustrated that the difference between auditory neutral and auditory congruent (*p* = 0.021) was significant. However, the difference between the auditory incongruent and auditory congruent (*p* = 0.19), as well as the auditory incongruent and auditory neutral (*p* = 0.53) was not significant. Tukey tests also showed that the difference between visual incongruent and visual congruent (*p* < 0.001), as well as visual incongruent and visual neutral (*p* < 0.001) was significant. Nevertheless, and as in Experiment 1, no significant difference between visual neutral and visual congruent conditions (*p* = 0.15) was observed.

#### Response Error

Response errors were analyzed with the same ANOVA design as for RT. There was a significant main effect of visual congruency on response errors, *F*_(2, 58)_ = 26.72, *p* < 0.001, $\eta_{p}^{2}$ = 0.48, while the effect of auditory congruency, *F*_(2, 58)_ = 0.89, *p* = 0.41, $\eta_{p}^{2}$ = 0.03, and the interaction of visual x auditory congruency, *F*_(4, 58)_ = 1.69, *p* = 0.15, $\eta_{p}^{2}$ = 0.06, were not significant. *Post-hoc* Tukey tests on visual congruency illustrated significant differences between the visual incongruent and visual neutral (*p* < 0.001), as well as the visual incongruent and visual congruent (*p* < 0.001) conditions. No difference in terms of response errors was observed between visual neutral and visual congruent (*p* = 0.18).

#### Distributional Analysis of Reaction Time

**Figure 5:**
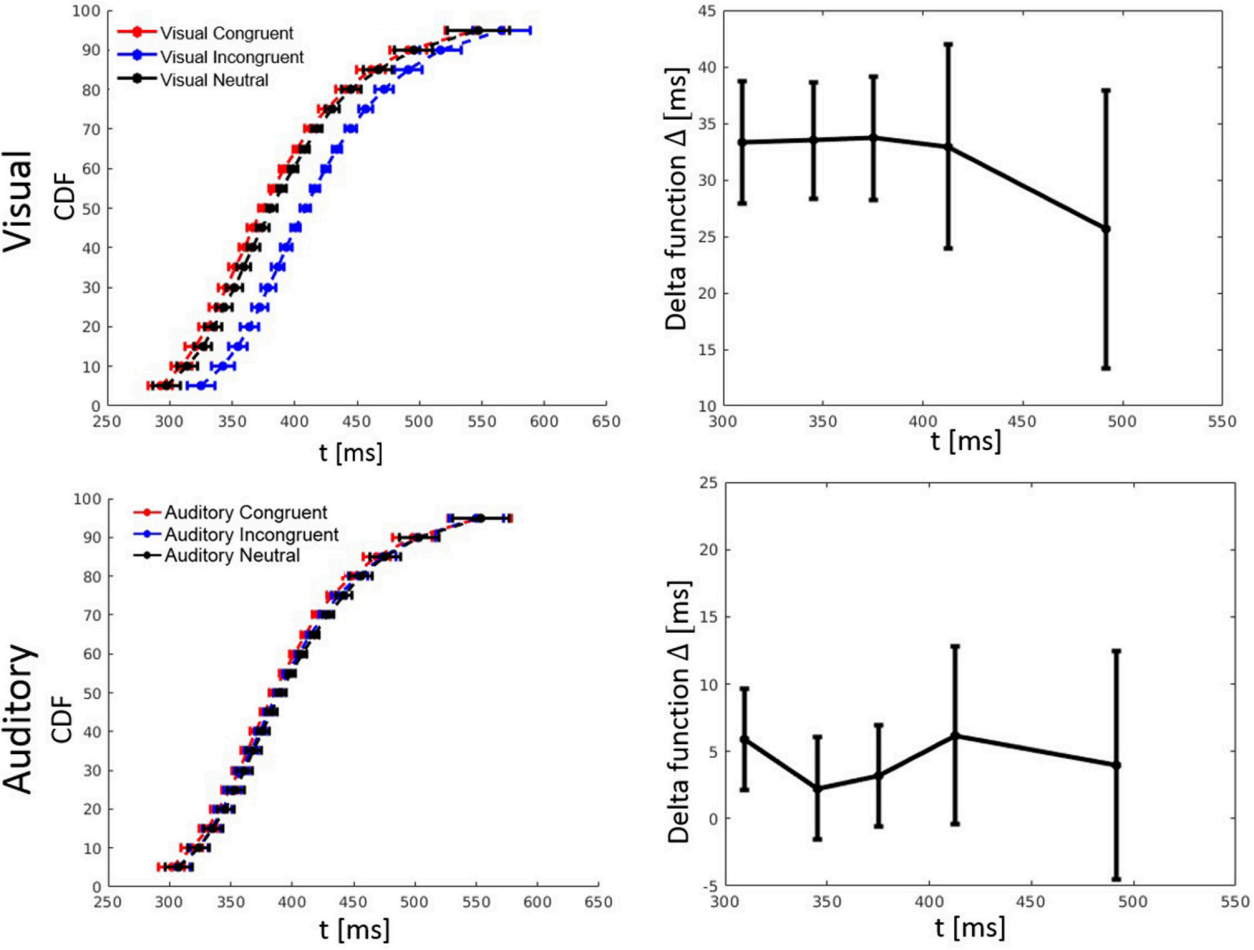
Cumulative distribution functions (CDFs) and delta (Δ) functions for percentiles (5, 10, 15, …, 95%) in Experiment 2. Error bars show 95% confidence intervals and are calculated according to Morey (2008). Each of the visual (auditory) CDFs was calculated as the average over all auditory (visual) congruency conditions. For example, the visual congruent CDF is the average of CVCA, CVNA, and CVIA conditions. Delta functions show the difference between the congruent and incongruent CDFs as a function of response time.

RT percentiles were estimated and analyzed as in Experiment 1 by a three-way ANOVA with factors percentile, visual congruency, and auditory congruency. The main effect of percentile, *F*_(4, 116)_ = 292.56, *p* < 0.001, $\eta_{p}^{2}$ = 0.91, the effect of auditory congruency, *F*_(2, 58)_ = 5.48, *p* = 0.007, $\eta_{p}^{2}$ = 0.16, and the effect of visual congruency, *F*_(2, 58)_ = 90.36, *p* < 0.001, $\eta_{p}^{2}$ = 0.76, were all significant. The three-way visual congruency x auditory congruency x percentile interaction, *F*_(16, 464)_ = 0.38, *p* = 0.99, $\eta_{p}^{2}$ = 0.01, and all of the two-way interactions were not significant. Figure 5 shows the CDFs for congruent, neutral, and incongruent conditions for both the visual and auditory modality. It also illustrates how the delta function decreases with an increase of RT.

#### Analysis of Conditional Accuracy Functions

CAFs were calculated similarly to the first experiment. A three-way ANOVA with factors bin, visual congruency, and auditory congruency showed a significant effect of visual congruency, *F*_(2, 58)_ = 8.23, *p* = 0.001, $\eta_{p}^{2}$ = 0.22. In contrast, the effect of auditory congruency, *F*_(2, 58)_ = 0.54, *p* = 0.59, $\eta_{p}^{2}$ = 0.02, and bins, *F*_(4, 116)_ = 0.48, *p* = 0.75, $\eta_{p}^{2}$ = 0.02, were not significant. The visual congruency x auditory congruency interaction was significant, *F*_(4, 116)_ = 2.51, *p* = 0.046, $\eta_{p}^{2}$ = 0.08. However, the three-way bin x visual congruency x tactile congruency interaction, *F*_(16, 464)_ = 1.06, *p* = 0.40, $\eta_{p}^{2}$ = 0.04, and all other two-way interactions were not significant.

### Discussion

The second experiment investigated how simultaneous task-irrelevant visual and task-irrelevant auditory information affects visual decisions. Exactly as in Experiment 1, task-irrelevant visual information evoked pronounced congruency effects on RT and response errors. However, the effects of the task-irrelevant auditory information were less pronounced than the effects of tactile information in Experiment 1. Although there was a significant effect of auditory congruency on RT, further analysis showed that this effect was due to especially slow responses in the neutral compared to the congruent condition. No significant difference between the congruent and the incongruent auditory condition was observed. The results regarding response errors indicate that task-irrelevant auditory information did virtually not affect the accuracy of the visual decisions.

Taken together, task-irrelevant visual information significantly affected RT and response errors of the visual decisions, which is in line with the typical Simon effect and the results of the first experiment. The lack of congruency effect of the task-irrelevant auditory information presumably suggests that the influence of auditory spatial information on visual information processing is rather limited in the presence of visual spatial information. This is in line with many studies suggesting that the visual modality dominates the auditory one in processing spatial information (Howard and Templeton, 1966; Welch and Warren, 1980; Bertelson and Radeau, 1981; Slutsky and Recanzone, 2001).

### Modeling

Similar to the previous models (Luce, 1986; Ulrich et al., 2015), total RT is assumed to be the sum of two parts (RT = D+R), that is, the duration of the decision process (D), and the duration of residual processes (R), which represent the duration of all processes besides the decision process. It is also assumed that the congruency of the stimuli only affects the duration of D and not of R. Within DMC, the decision process is modeled as a standard Wiener diffusion process. Specifically, the state *X*(*t*) of the decision process at time is *t* regarded as a superimposed Wiener process, that is, *X*(*t*) = *X*_*c*_(*t*)+*X*_*a*_(*t*), where *X*_*c*_(*t*) denotes a controlled process and *X*_*a*_(*t*) an automatic process. The superimposed process accumulates until it hits either the upper (correct) decision boundary (*b* > *0*) or the lower (incorrect) decision boundary (–*b*).

According to the original version of DMC, the controlled process can be described by the following stochastic difference equation

(1) $$\begin{array}{r} X_{c}\left(t+\Delta t\right)=\;X_{c}\left(t\right)+\;\mu_{c}\left(t\right)\cdot \;\Delta t+W_{c}\left(t\right)\cdot \sigma_{c}\cdot \sqrt{\Delta t} \end{array}$$

where *X*_*c*_(*t*) denotes the state of the controlled process at time *t*. *W*_*c*_(*t*)is the standard Wiener diffusion process (mean = 0, and variance = 1), σ_*c*_ indicates the diffusion constant, and μ_*c*_(*t*) is the time-independent drift rate of the controlled process, that is, μ_*c*_(*t*) = μ_*c*_. Likewise, the automatic process is given by

(2) $$\begin{array}{r} X_{a}\left(t+\Delta t\right)=\;X_{a}\left(t\right)+\;\mu_{a}\left(t\right)\cdot \;\Delta t+W_{a}\left(t\right)\cdot \sigma_{a}\cdot \sqrt{\Delta t} \end{array}$$

where *W*_*a*_(*t*) is a Wiener diffusion process, with diffusion constant σ_*a*_. The drift rate of the automatic process μ_*a*_(*t*) is time-dependent.

Here we extend DMC in order to fit the data from the multimodal Simon task studied in Experiments 1 and 2. In the multimodal DMC (MDMC), two (or more) automatic processes superimpose on the controlled process to form the decision process: *X*(*t*) = *X*_*c*_(*t*)+*X*_*a*1_(*t*)+*X*_*a*2_(*t*). *X*_*c*_(*t*) denotes again a standard Wiener diffusion process with the constant time-independent drift μ_*c*_(*t*) = μ_*C*_. The time course of an automatic process is modeled as a pulse-like rescaled Gamma distribution, *X*_*a*_(*t*), with shape parameter *a* = 2 and the free scale parameter τ. The parameter *A* corresponds to the maximum of this pulse-like function. Thus, the time course of the expected mean of the automatic process is given by (cf. Ulrich et al., 2015)

(3) $$\begin{array}{r} E\left[X_{a}\left(t\right)\right]=A\cdot e^{-\frac{t}{\tau }}\cdot {\left[\frac{t\cdot e}{\left(a-1\right)\cdot \tau }\right]}^{a-1} \end{array}$$

and thus the time-dependent drift rate μ_*a*_(*t*) of the automatic process is given by the first derivative of *E*[*X*_*a*_(*t*)] with respect to time,

(4) $$\begin{array}{r} \mu_{a}\left(t\right)=A\cdot e^{-\frac{t}{\tau }}\cdot {\left[\frac{t\cdot e}{\left(a-1\right)\cdot \tau }\right]}^{a-1}\cdot \left[\frac{a-1}{t}-\frac{1}{\tau }\right]. \end{array}$$

**Figure 6:**
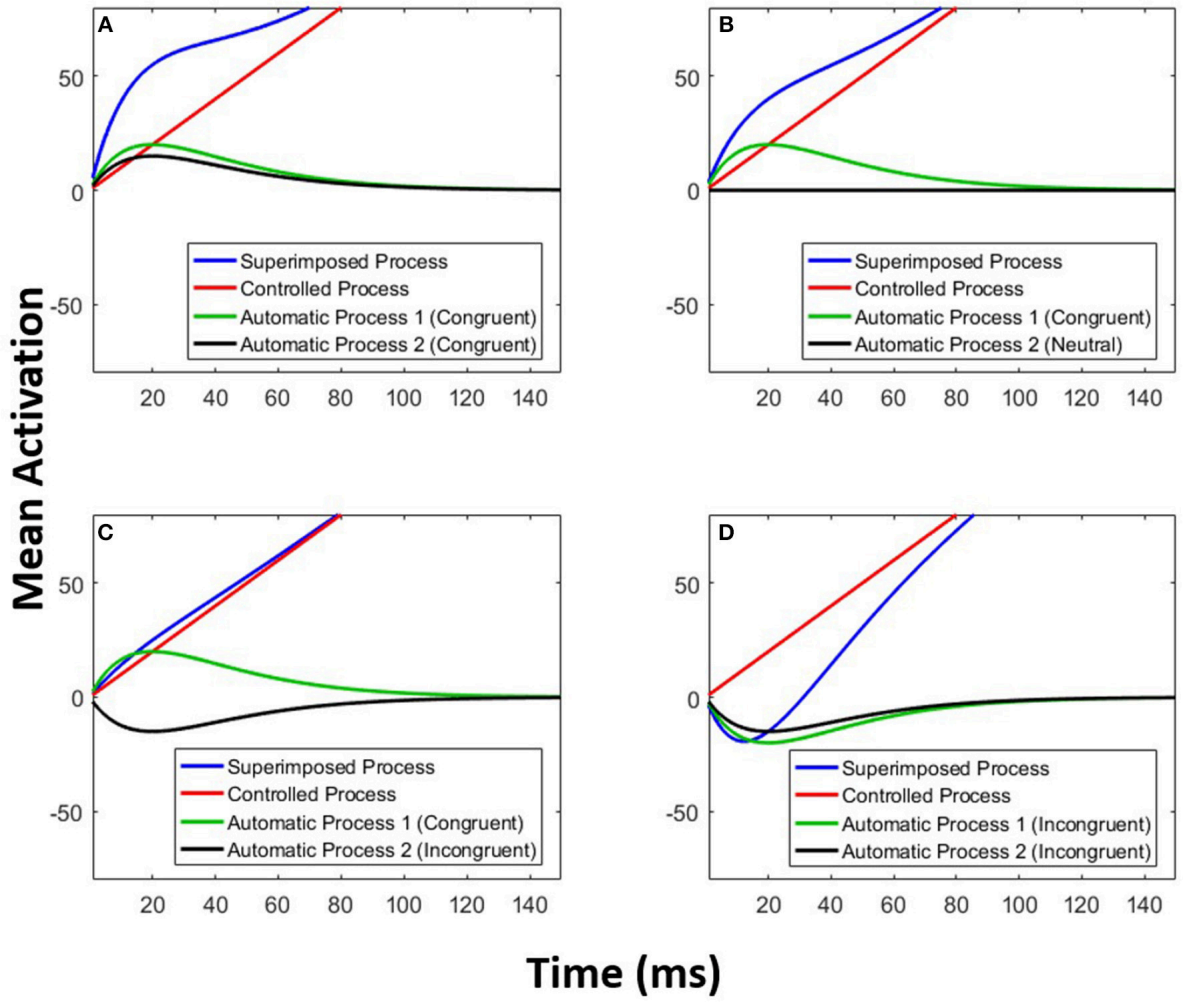
Multimodal DMC. The decision process (blue line) is a superimposition of a controlled process (red line) and two automatic processes (green and black lines). **(A)** Both of the automatic processes are congruent. **(B)** The first automatic process is congruent and the second one is neutral. **(C)** The first automatic process is congruent and the second one is incongruent. **(D)** Both of the automatic processes are incongruent.

The parameters *A* and τ are estimated for each of the two automatic processes. Figure 6 exemplifies the architecture of MDMC. The expected decision process *E*[*X*(*t*)] (blue line) is modeled as the sum of the expected controlled process *E*[*X*_*c*_(*t*)] (red line) and two expected automatic processes *E*[*X*_*a*1_(*t*)] and *E*[*X*_*a*2_(*t*)] (black and green lines). A congruent automatic process is represented by a positive (i.e., *A*>0) pulse-like function (e.g., Figure 6A: both of the automatic processes are congruent), and an incongruent automatic process is represented by a negative (i.e., *A* < 0) pulse-like function (e.g., Figure 6D: both of the automatic processes are incongruent). It is assumed that the neutral automatic process does not affect the decision process (Figure 6B: black line). The trial-to-trial variability of the starting point is modeled by random samples from a beta distribution, with the free parameter α, supported on the bounded interval [-b, b], where b is the decision boundary.

We fitted two variants of MDMC to the data, one as is described so far, and one with a faster processing of neutral visual information. Previous studies mentioned that a visual stimulus at the center of field of view (FOV) benefits from faster retinal processing in contrast to a stimulus presented to the left or to the right of the center (fixation point) (Osaka, 1976). For example, presenting a stimulus by 5–10° degree nasal or temporal from the fovea typically increases RT by 10–20 ms (Rains, 1963). This phenomenon motivates an extension of MDMC with a separate mean residual process time for neutral visual information. This version of the MDMC model is called FN-MDMC (Faster Neutral visual-Multimodal DMC).

#### Fitting Criteria

The fitting procedure was similar to the method described by Hübner (2014) and also Servant et al. (2016). The MDMC was fitted to the CAFs and the CDFs for each of the nine congruency conditions. There were five CAF bins (0–20, 20–40, 40–60, 60–80, 80–100%), and five CDF quantiles (0.1, 0.3, 0.5, 0.7, 0.9) for each given congruency condition. MDMC predictions were generated using Monte Carlo simulations (Metropolis and Ulam, 1949) with a step size of Δ*t* = 1 *ms*, and a constant diffusion constant of σ = 4 *ms* for the superimposed process, similar to Ulrich et al. (2015). The following function was employed to fit the model to the data

(5) $$\begin{array}{r} G^{2}=\;2\sum_{c\;=\;1}^{9}N_{c}\sum_{i\;=\;1}^{10}|p_{ci}\log\left(\frac{p_{ci}}{\pi_{ci}}\right)| \end{array}$$

where *p*_*ci*_ and π_*ci*_ denote the observed and the predicted proportion of responses, respectively. The index *c* indicates the congruency condition, and the summation over the *i* includes both CAFs (five bins) and CDFs (five bins). *N*_*c*_ is the number of trials per congruency condition. Fifty thousand trials were simulated for each minimization call in each of the congruency conditions. The *G*^2^ criterion was minimized using the MATLAB implementation of the SIMPLEX (Lagarias et al., 1998) method. Since SIMPLEX is sensitive to the choice of initial values, the fitting procedure was repeated with different sets of initial values in order to ensure the stability of the resulting estimates.^1^

#### Fitting MDMC

MDMC was fitted to the aggregated experimental data over all participants using the aforementioned criteria. We fitted the model to the averaged data of all participants because the data of individual participants are typically noisy and may be prone to outlier RTs. Especially if trial numbers are rather small, it is difficult to identify the best fitting model parameters for individual participant data. Even though a previous study showed a virtually negligible difference between fitting DMC to individual data and to average group data (Servant et al., 2016), it should be highlighted that fitting data to group averages neglects interindividual variability and thus may result in distortions of parameter estimates (e.g., Estes and Maddox, 2005; Cohen et al., 2008). Nevertheless, the results of model fits to individual data are available in the Supplementary Material. Moreover, raw data and the complete Matlab code for model fitting are available online via the Open Science Framework (Mahani, 2018).

**Figure 7:**
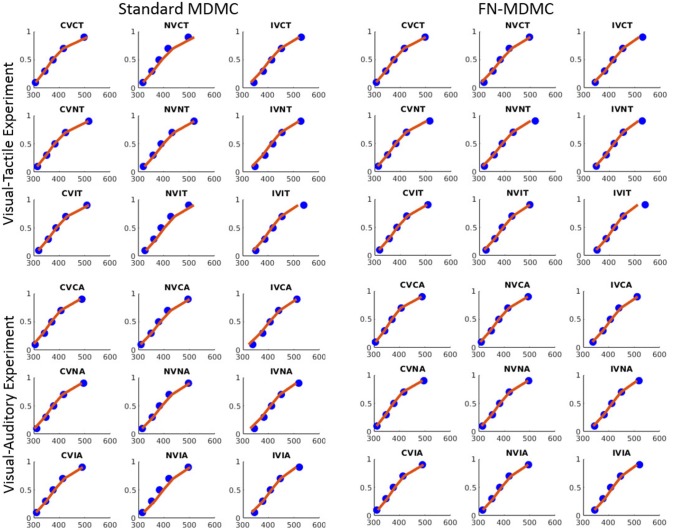
Experimental data and model predictions of CDFs for both experiments. Blue dots show the experimental data and red lines show the model predictions. Both models provide a reasonable fit of the experimental data, however, FN-MDMC fits slightly better than MDMC.

**Figure 8:**
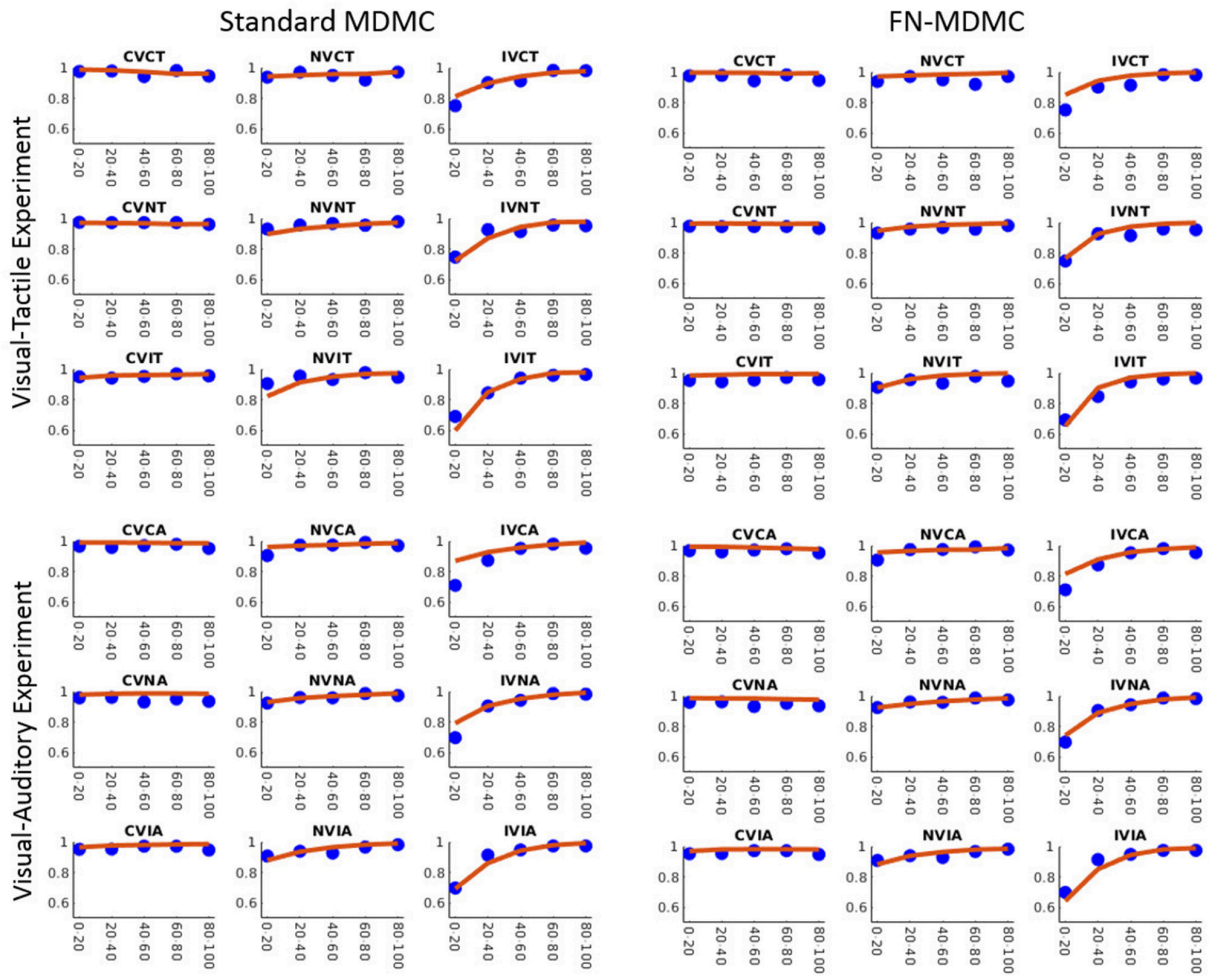
Observed results and model predictions of CAFs for both experiments, across all congruency conditions, and for both variants of the model. Blue dots show the experimental data and red lines show the model predictions. The model appropriately predicts the experimental data except for small proportions of the incongruent visual conditions.

**Figure 9:**
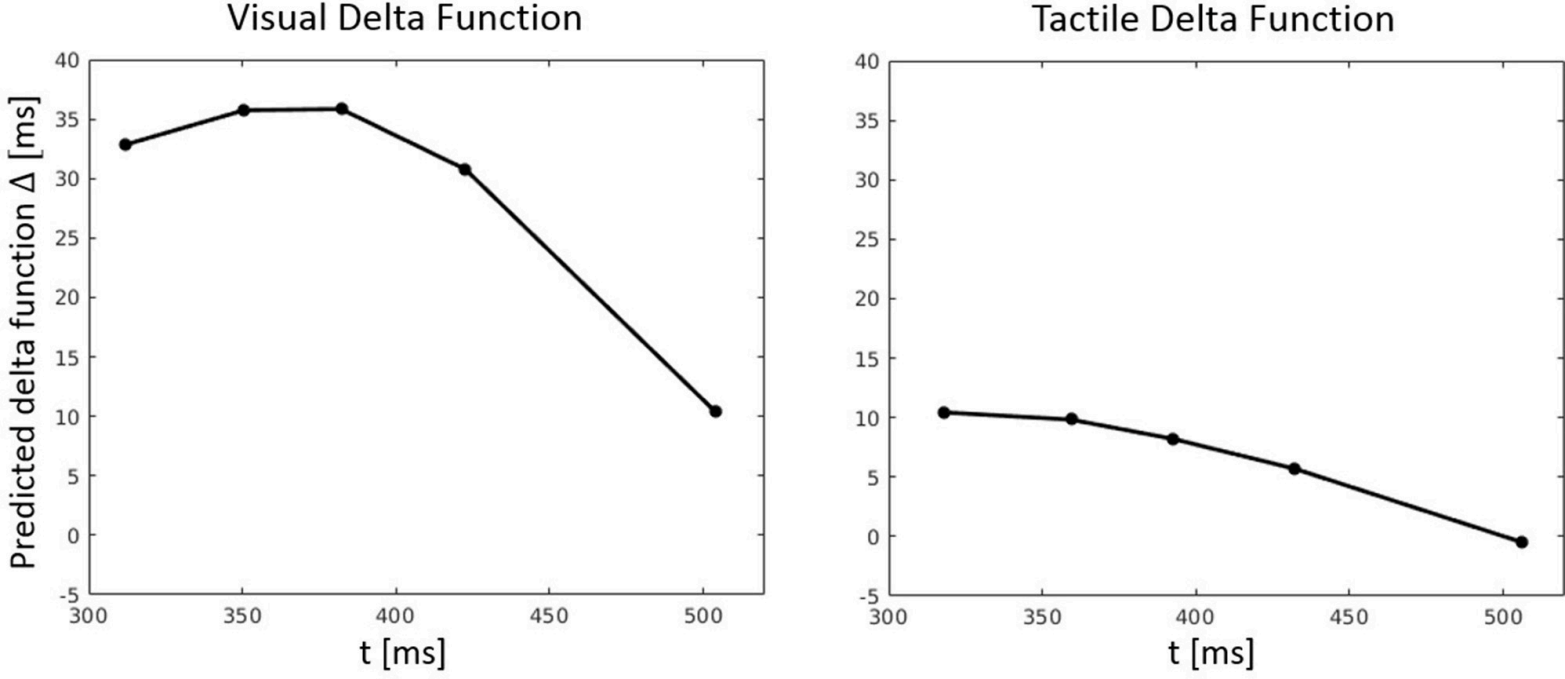
Predicted delta (Δ) functions by FN-MDMC for the visual-tactile experiment. Delta functions show the difference between the congruent and incongruent CDFs as a function of response time.

**Figure 10:**
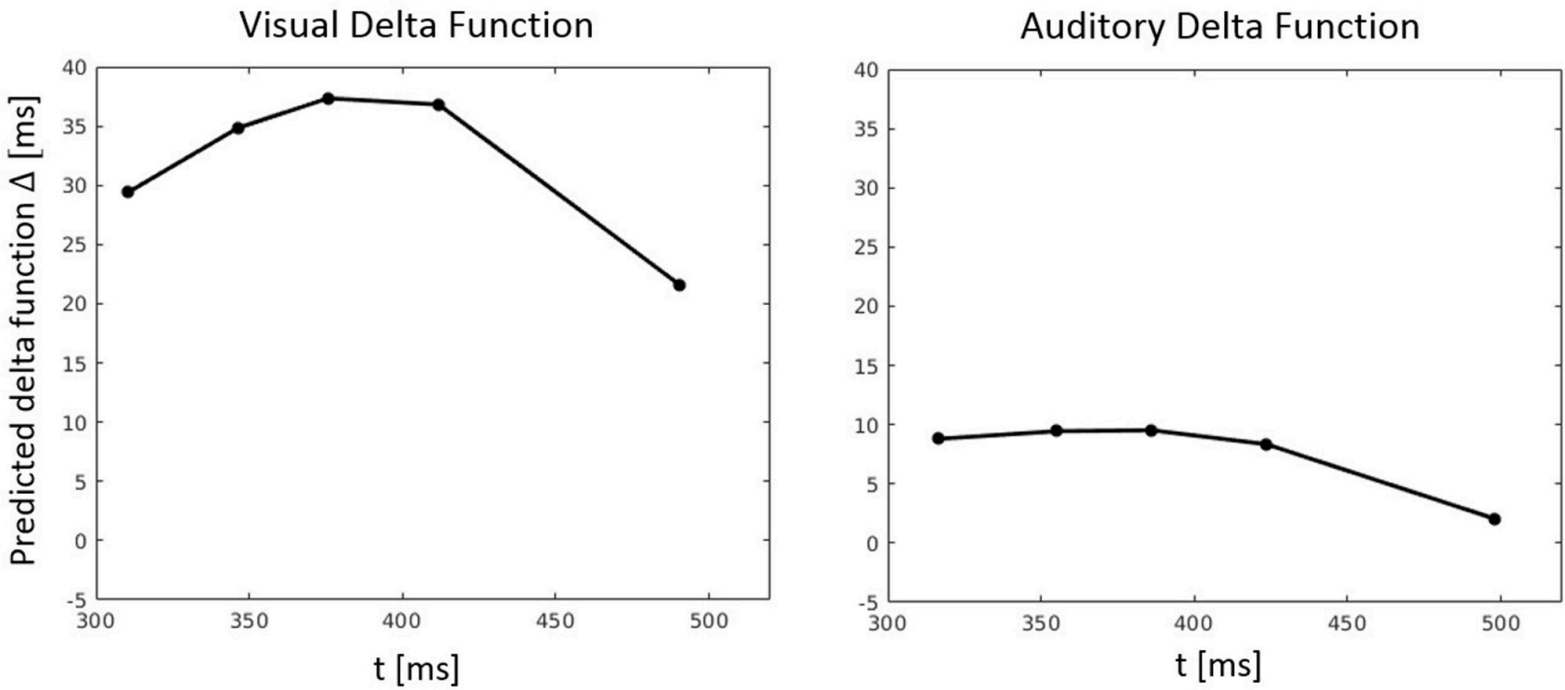
Predicted delta (Δ) functions by FN-MDMC for the visual-auditory experiment. Delta functions show the difference between the congruent and incongruent CDFs as a function of response time.

Figures 7, 8 show the results for both CDFs and CAFs in all congruency conditions of both experiments. Figures 9, 10 also depict predicted delta functions of the FN-MDMC model for both experiments. In general, MDMC provides a reasonable fit of the experimental data. However, FN-MDMC fits slightly better than MDMC. Observing more errors for faster RTs is a common pattern in Simon tasks and this is especially bold in the incongruent visual conditions of the present experiments. MDMC captures this pattern relatively well, with only a few small deviations (cf. Figure 8). Table 1 contains the estimated parameters for both the visual-tactile and the visual-auditory task, and for both variants of the model. This table also provides the average of *G*^2^ for 1,000 simulations given the best parameters for each model. Similar to Servant et al. (2016), we compared MDMC and FN-MDMC by a *BIC* statistic that penalizes models based on the *G*^2^ and number of free parameters *f*:

(6) $$\begin{array}{r} BIC=\;G^{2}+f\;log\sum_{i\;=\;1}^{n}n_{i} \end{array}$$

**Table 1:** Parameter estimates for the model fit of MDMC and FN-MDMC to the results of the visual-tactile (V-T, Experiment 1) and visual-auditory (V-A, Experiment 2) tasks.

**Task**	**Model**	**μ_*R*_**	**μ_*RN*_**	**σ_*R*_**	**α**	***b***	**μ_*C*_**	***A*_*V*_**	**τ_*V*_**	***A*_*T*/*A*_**	**τ_*T*/*A*_**	***G*^2^**	***BIC***
V-T	MDMC	313	–	33.4	3.1	54.6	0.52	13.4	39.0	6.1	28.5	126.1	176.5
	FN-MDMC	317	303	36.8	2.9	64.1	0.66	19.8	38.5	7.1	28.2	114.5	170.5
V-A	MDMC	311	–	40.5	2.7	57.7	0.62	15.1	51.4	6.5	35.5	136.2	186.6
	FN-MDMC	315	302	35.3	3.5	55.6	0.58	16.7	46.6	5.3	32.0	98.6	154.6

*Unit of measurement for μ_R_, μ_RN_, σ_R_ is the millisecond (ms), while the unit of μ_C_ is $\frac{\text{1}}{ms}$*.

We compared the fits of MDMC and FN-MDMC using the paired-sample permutation test across 1,000 simulated *G*^2^ and *BIC* values with 50,000 permutations. In the visual-tactile experiment, both the *G*^2^ and *BIC* of FN-MDMC were significantly lower than *G*^2^ and *BIC* of MDMC (*p*s < 0.001). The same result was obtained for the visual-auditory experiment, that is, *G*^2^ and *BIC* of FN-MDMC were also significantly lower than *G*^2^ and *BIC* of MDMC (*p*s < 0.001). Table 1 shows the average of simulated *G*^2^ and *BIC* values of the two experiments.

Note that μ_*R*_ and σ_*R*_ represent the mean and standard deviation of the residual process time, respectively. However, the mean of the residual process time for the neutral visual condition is given by μ_*RN*_ in the FN-MDMC model. α and *b* correspond to the shape and decision boundary of the starting point distribution, respectively. μ_*C*_ is the drift rate of the controlled process. *A* and τ are the parameters of the automatic process for each modality (see Equation 3). The only difference between MDMC and FN-MDMC is the addition of the parameter μ_*RN*_ in the latter case to enable a direct assessment of the effect of μ_*RN*_ on the goodness of fit. In both FN-MDMC models the shorter mean residual process time for the visual neutral condition (μ_*RN*_) compared to the mean residual process time of the other congruency conditions (μ_*R*_) results in a smaller fitting error (cf. Table 1). This result is consistent with the phenomenon that visual stimuli presented at the fovea benefit from faster processing and the size of this effect agrees with the typical speed benefit for foveal processing (Rains, 1963).

**Figure 11:**
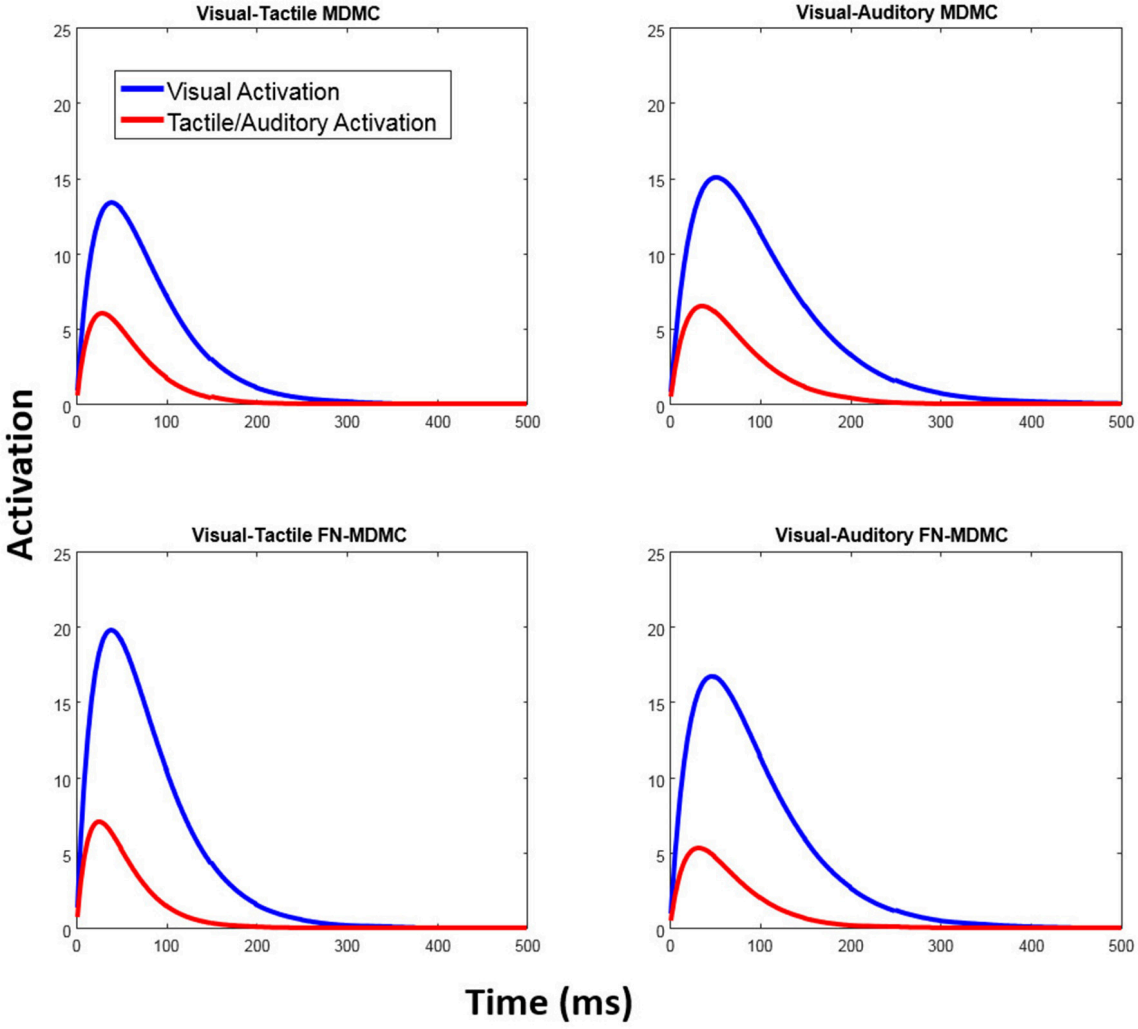
Automatic activation processes of the fitted models. In both models, the peak activation of the visual automatic process is higher than the peak activation of the automatic tactile/auditory process and thus reflects the relatively strong influence of visual-spatial task-irrelevant information.

Table 1 also reveals that in all models the peak activation of the visual automatic process (*A*_*V*_) is higher than the peak activation of the tactile/auditory automatic process (*A*_*T*/*A*_). This result points to a relative dominance of visual stimuli over tactile/auditory stimuli (see Figure 11).

## General Discussion

Numerous studies have suggested that task-irrelevant information affects the task-relevant decision processes in speeded RT tasks. The standard Simon task assesses the influence of task-irrelevant information on the processing of task-relevant information within the visual modality. In the present study, we investigated whether additional task-irrelevant information from the tactile modality (Experiment 1) or from the auditory modality (Experiment 2) would also influence the processing of visual information. The experiments were theoretically motivated by an elaboration of DMC, which assumes that task-irrelevant information from different sense modalities superimpose. Specifically, this elaboration assumes that the contribution of task-irrelevant information from one modality does not affect the contribution of task-irrelevant information from the other modality. MDMC further assumes that this superimposed information spills over to the decision process. The MDMC provided a reasonable account for the results of the two experiments. As expected, the results of the two experiments revealed the classical Simon effect (i.e., a task-irrelevant influence of spatial visual stimulus position on RT and response errors). In Experiment 1 we also observed the influence of task-irrelevant tactile stimulation on RT and response errors of visual decisions. In Experiment 2 the task-irrelevant auditory information affected the RT of visual decisions, but not the response errors. Furthermore, there was no difference between the auditory congruent and auditory incongruent conditions, pointing to an unreliable effect of auditory stimulus location on the RT of visual decisions. Moreover, the observed delta functions, especially for the visual congruency conditions, are negative-going, thus indicating that the congruency effect decreases with increasing reaction time. Such negative-going delta functions have been repeatedly reported for the Simon task (for an overview, see Schwarz and Miller, 2012).

Our findings also corroborate the robust phenomenon showing that task-irrelevant spatial visual information affects visual decisions. Even though there is a large number of studies on the effects of task-irrelevant information on non-visual decisions (MacLeod, 1991; Lu and Proctor, 1995; Dolk et al., 2014), so far, no one studied the effects of simultaneous task-irrelevant tactile and visual information on non-spatial visual decisions. However, several studies reported cross-modal effects of touch on visual perception (Macaluso et al., 2002; Diederich et al., 2003; Ossandón et al., 2015). Therefore, we expected to observe an influence of task-irrelevant tactile information on RT and response errors for visual decisions, and the results of Experiment 1 are consistent with these expectations.

In Experiment 2, however, the lack of a clear effect of auditory stimulation on visual decisions was rather unexpected in the light of previous studies (Simon and Craft, 1970; Donohue et al., 2013; Schupak et al., 2015). For example, Simon and Craft showed that task-irrelevant auditory information can influence visual decisions in a Simon task. However, in this study, auditory stimulation was not accompanied by simultaneous task-irrelevant visual stimulation, as in the present Experiment 2. In fact, several other studies have reported the lack of or small effects of task-irrelevant auditory information on visual decisions in co-presence of both visual and auditory spatial information (Howard and Templeton, 1966; Welch and Warren, 1980; Bertelson and Radeau, 1981; Slutsky and Recanzone, 2001). These observations suggest that the effect of task-irrelevant visual information on visual decisions is much stronger than the effect of task-irrelevant auditory information when simultaneous visual and auditory stimulation is provided. Thus, the relatively small effect of task-irrelevant auditory stimulation in the present Experiment 2 might be attributed to the fact that the auditory stimulus did not carry task-relevant information and was accompanied by visual-spatial stimulation. This is also reflected in the fitted parameters of the MDMC and FN-MDMC, as a relatively small peak of the automatic activation corresponding to the auditory compared to the visual stimulation (cf. Figure 11, bottom row, and Table 1).

The performance of participants in the neutral conditions revealed a rather surprising pattern. Intuitively, one might expect that the mean RT in the neutral condition is just the average of the RTs in the congruent and incongruent conditions, if the influences of inhibition and facilitation are equally effective. Contrary to this expectation, neither RT nor response errors did significantly differ between the visual neutral and visual congruent conditions. Interestingly, MDMC assumes that the effects of inhibition and facilitation on the decision process are symmetrical (i.e., automatic activation in incongruent trials favors the wrong response to the same amount as automatic activation in congruent trials favors the correct response). Nonetheless, it can be shown that this symmetry need not necessarily manifest itself at the level of mean RT. It must be admitted, however, that the deviation from symmetry was so large that it cannot be captured quantitatively by MDMC.

There is at least one explanation for this asymmetry effect. One may generally refute the idea that it is possible to introduce a true neutral condition in conflict task paradigms in order to reveal the contributions of interference and facilitation, as previous studies with such baseline conditions suggest (Simon and Acosta, 1982). For example, a neutral stimulus presented at the fixation point may benefit from retinal processing in contrast to stimuli presented in the periphery, that is, to the left or to the right of the fixation point (Slater-Hammel, 1955; Osaka, 1976). Within MDMC, this would simply mean that the residual process operates faster in the visual neutral condition than in both the congruent and incongruent ones. Hence, we have investigated this proposal by extending the MDMC to FN-MDMC. The FN-MDMC indeed provides a better model fit than the standard MDMC and, as one might expect, corroborates a faster residual process for neutral visual information. Specifically, the FN-MDMC reveals that when a visual stimulus is presented at the fovea, processing time is ~10–15 ms faster than in the periphery. This finding is consistent with simple reaction time results from a previous study (Rains, 1963).

The asymmetrical effect produced by tactile stimulation in Experiment 1 is probably more surprising than the aforementioned asymmetrical congruency effect in the visual modality. Here we observed that RTs in the neutral tactile condition were not significantly different from those in the incongruent tactile condition, although response errors were about the same as in the congruent tactile condition. One can only speculate about the reasons for this surprising pattern of results. One reason may be that tactile stimulation along the body's median sagittal plane takes more time to process than along the body's horizontal plane. Accordingly, the residual process within MDMC should take more time for central than for peripheral tactile stimuli. Unfortunately, this account cannot address the difference in response errors. Another speculation is that there is a tradeoff between speed and accuracy within the tactile modality, which seems difficult to address within the present version of MDMC. Thus, providing a comprehensive interpretation of the tactile neutral condition is difficult. However, the results of the tactile neutral condition show that tactile stimulation cannot be ignored even if it provides task-irrelevant, modality-irrelevant, and neutral information.

In the present work, MDMC was fitted to average data. Model fits to individual data are presented in the Supplementary Material. The parameter estimates of both approaches are reasonably similar. Nevertheless, we preferred model fits to averaged data in the present case in order to reduce not only the computational complexity and effort, but also to minimize the influence of spurious responses that may render individual datasets noisy. Future efforts should be directed toward overcoming these limitations, for example, with the Approximate Bayesian Computation approach (Turner and Van Zandt, 2018).

In conclusion, the present study examined crossmodal congruency effects within the classical visual Simon task. In Experiments 1 and 2, the spatial position of task-irrelevant tactile and auditory stimulation, respectively, varied orthogonally with the spatial position of the relevant visual information. MDMC provided a reasonable account of the observed RT data and response errors. This model suggests that task-irrelevant activation combines additively across modalities before the summed automatic activation spills over to the processing of task-relevant information. MDMC's predictions, however, were suboptimal with regard to the neutral conditions. One reason for this suboptimal prediction is that the neutral conditions may not provide an ideal baseline for assessing the respective contributions of facilitation and inhibition through congruence and incongruence within the Simon task, a conclusion that receives support from other experimental work. In fact, the model fit was improved by an extension of MDMC, which incorporates faster residual processing time for foveally presented (neutral) visual stimuli than for peripherally presented (congruent and incongruent) stimuli. Importantly, this model extension acknowledges potential differences in processing latency according to stimulus location within the visual field, but does not change our main conclusion that superimposed automatic activation from multiple task-irrelevant information sources may overlap controlled stimulus processing. Therefore, MDMC offers a novel framework for understanding such multisensory processing in conflict tasks and thus advances our understanding of how information from different sensory modalities is processed and integrated, which is a core issue in neurocognitive sciences (e.g., Miller, 1982; Stein and Stanford, 2008).

## Author's Note

This study was conducted in the laboratory of RU while the first author was on sabbatical leave at the Department of Psychology, University of Tübingen. Raw data and Matlab code used for model fitting are available via Open Science Framework (Mahani, October 29, 2018, doi: 10.17605/OSF.IO/EWSJD) and via GitHub (https://github.com/manmahani/mdmc/). Correspondence concerning this article should be addressed to Mohammad-Ali Nikouei Mahani, Department of Psychology, University of Tübingen, Schleichstrasse 4, 72076 Tübingen, Germany, email address: nikouei@ut.ac.ir.

## Author Contributions

All authors contributed in the main manuscript text. M-AM and KMB collected the data. M-AM and RU contributed to the data analysis. RU has designed the study experiment and all authors reviewed the manuscript.

### Conflict of Interest Statement

The authors declare that the research was conducted in the absence of any commercial or financial relationships that could be construed as a potential conflict of interest.

## Footnotes

^1^To validate this parameter estimation procedure MDMC data were simulated with the estimated parameters reported in Table 1. Then, the recovery of the original parameters from these simulated data was assessed.
