# Supplementary material for: Multimodal Simon Effect: A Multimodal Extension of the Diffusion Model for Conflict Tasks
Source: Front Hum Neurosci. 2019 Jan 9;12:507. doi: 10.3389/fnhum.2018.00507 (PMC6333713; doi:10.3389/fnhum.2018.00507)
Supplement: Supplementary file 1 [file Data_Sheet_1.PDF]

Supplementary Material

Multimodal Simon Effect: A Multimodal Extension of the Diffusion Model for Conflict Tasks

Mohammad-Ali Nikouei Mahani*, Karin Maria Bausenhart, Majid Nili Ahmadabadi, and Rolf Ulrich

*** Correspondence:** Corresponding Author: nikouei@ut.ac.ir

1 Fitting MDMC and FN-MDMC to individual experimental results

The data of individual participants are usually very noisy with multiple local minima. This is mainly because the number of trials per condition is limited for each participant. Therefore, it is not always easy to find the absolute minimum. Moreover, individual data sets are distorted by RT outliers. Nevertheless, we have fitted MDMC and FN-MDMC to individual data sets. Figure 1 and Figure 2 show CDFs and CAFs in all congruency conditions of both experiments, averaged over individual fits. Figure 3 and Figure 4 depict the predicted delta functions by MDMC and FN-MDMC models for the visual-tactile and the visual-auditory experiment, respectively. Error bars in all figures illustrate the 95% confidence interval, which is computed according to Morey (2008). Finally, Table 1 gives parameter estimates averaged across the individual fits, along with the respective standard error of the mean (*SE*).

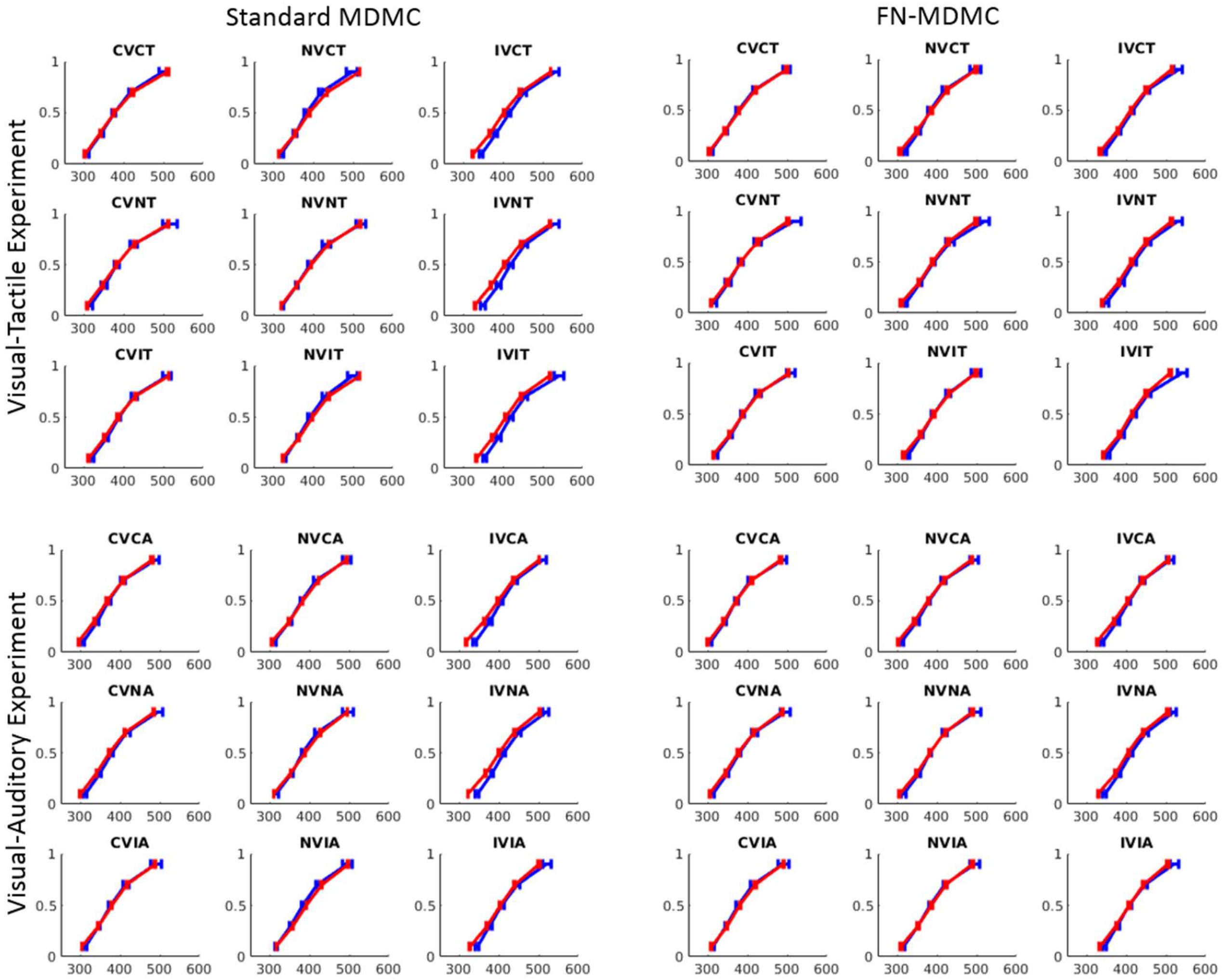

Figure 1. Experimental data and model predictions of CDFs for both experiments. Plots show the average of individual CDFs and individual fits and error bars show the 95% confidence interval, computed by Morey (2008). Blue lines show the experimental data and red lines show the model predictions.

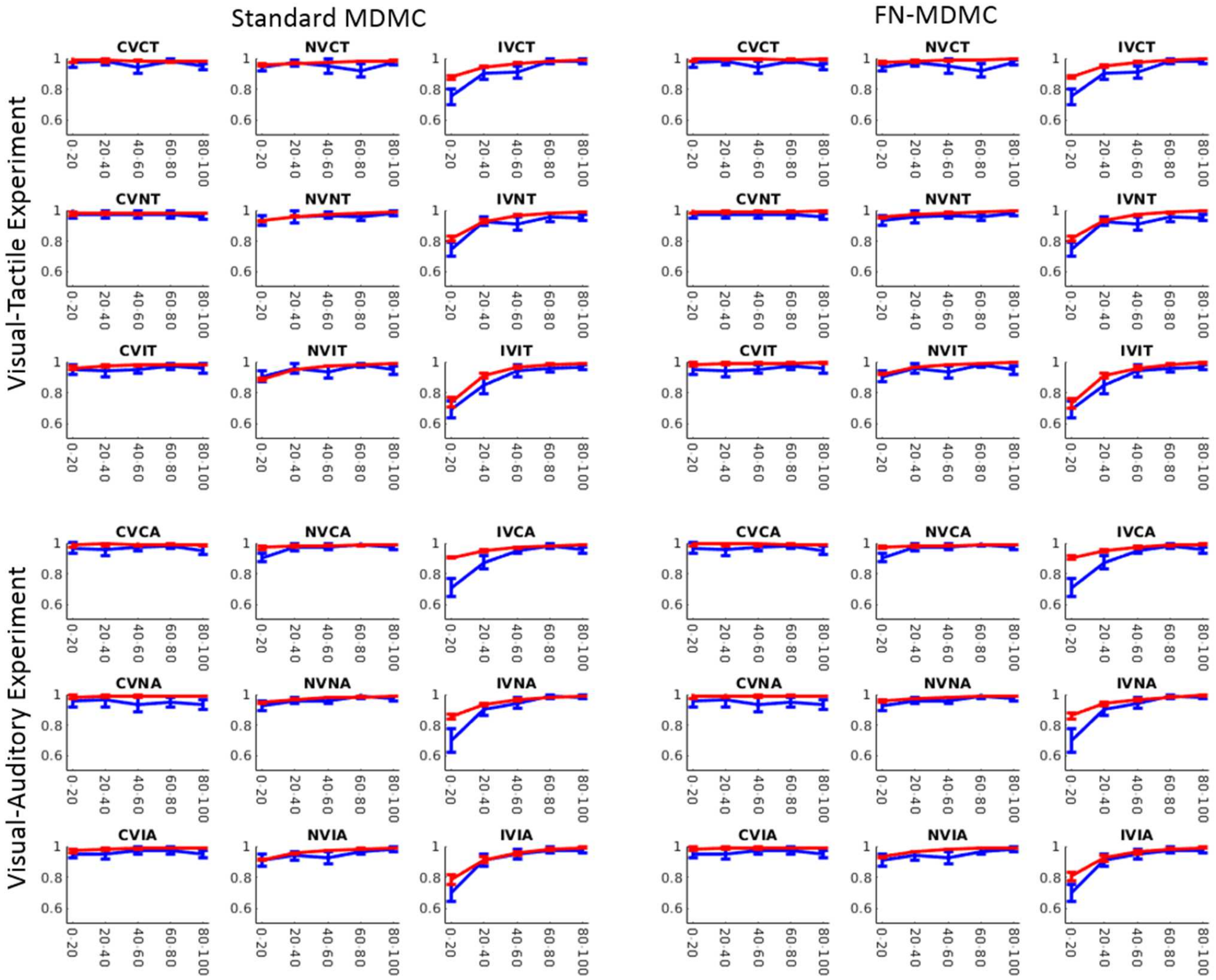

Figure 2. Observed results and model predictions of CAFs for both experiments. Plots show the average of individual CAFS and individual model fits. Blue lines show the experimental data and red lines show the model predictions.

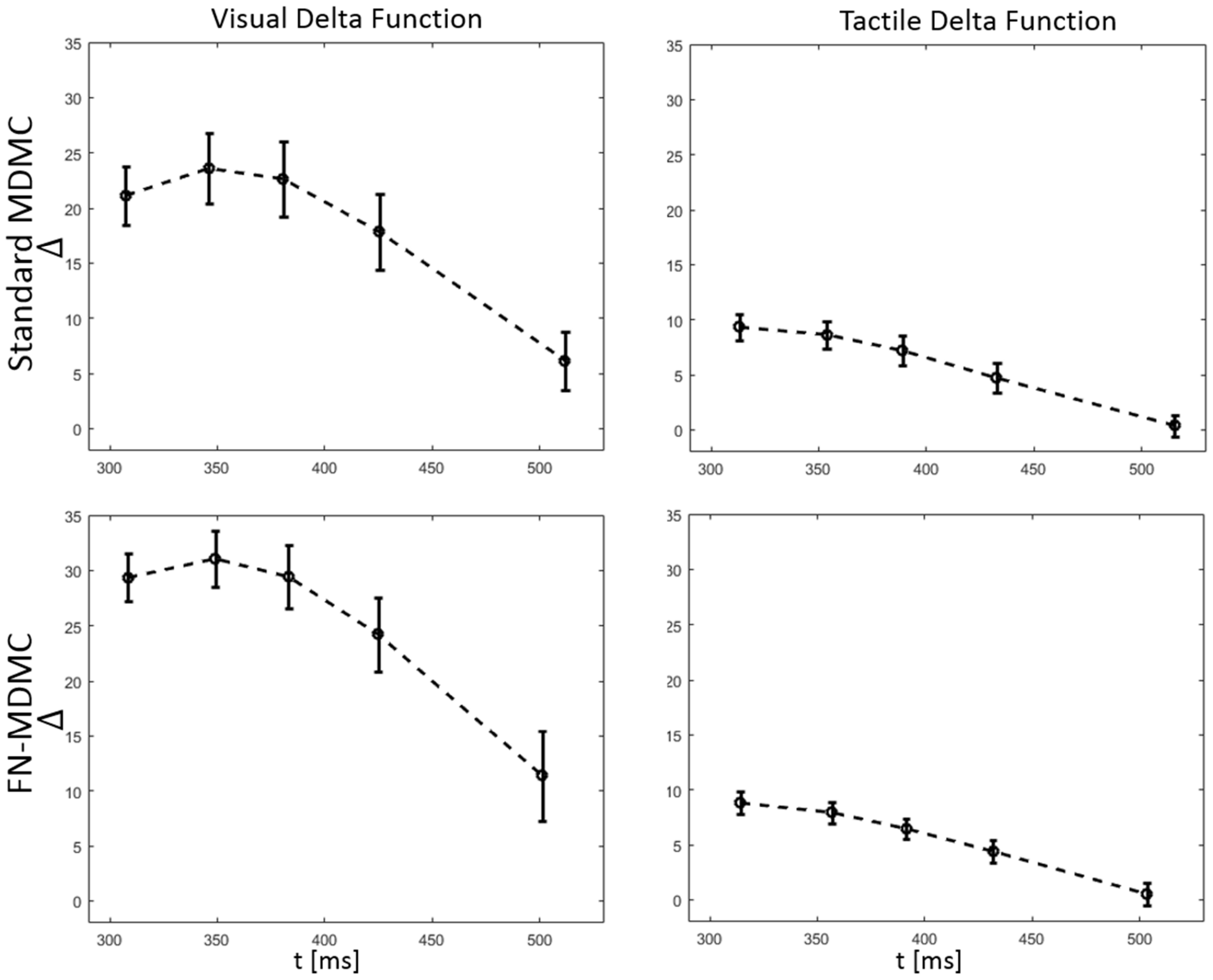

Figure 3. **Average of predicted delta (Δ) function by MDMC (top row) and FN-MDMC (bottom row) over individual fits for the visual-tactile experiment. Delta functions show the difference between the congruent and incongruent CDFs as a function of response time.**

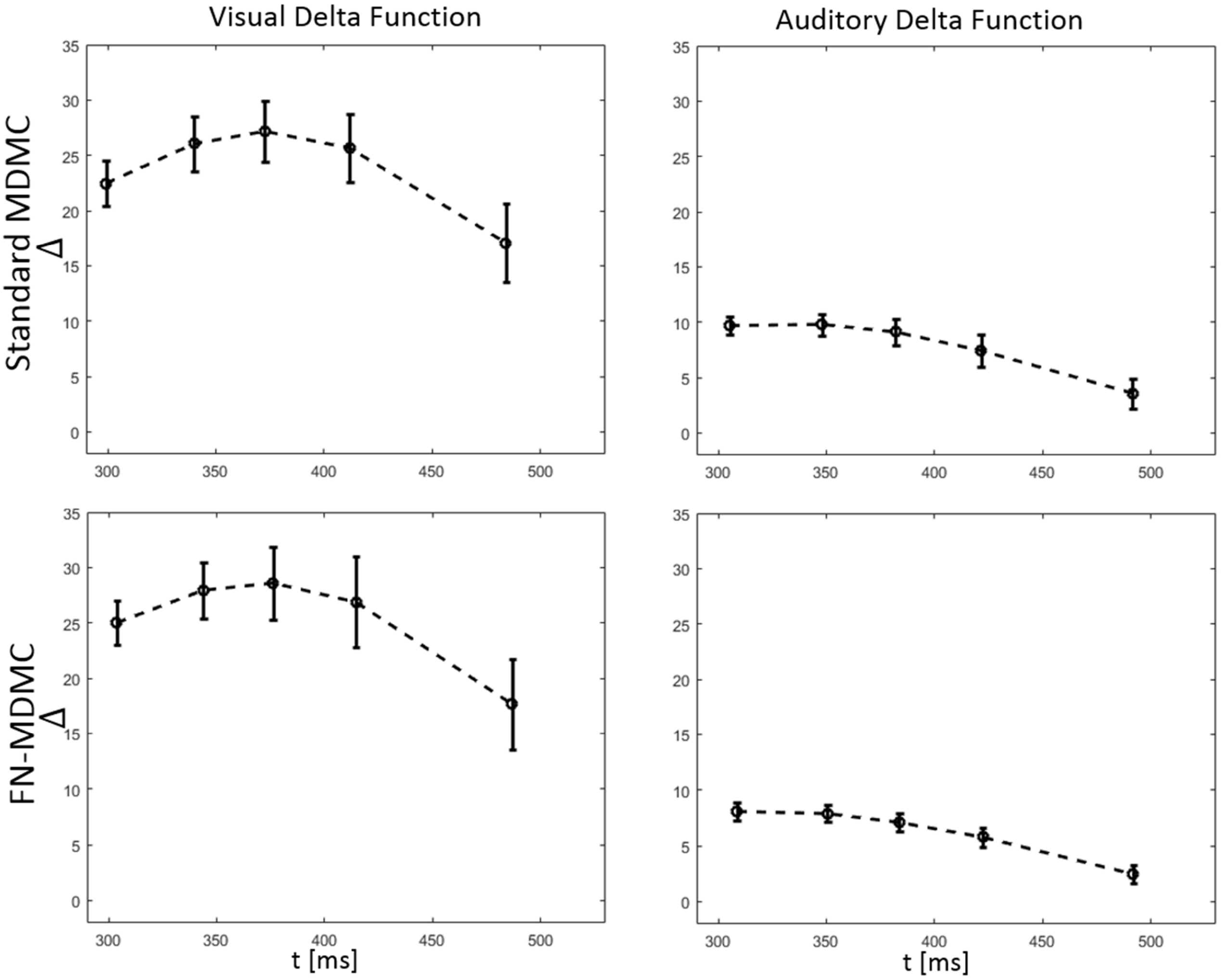

Figure 4. **Average of predicted delta (Δ) function by MDMC (top row) and FN-MDMC (bottom row) over individual fits for the visual-auditory experiment. Delta functions show the difference between the congruent and incongruent CDFs as a function of response time.**

Table 1.

Mean parameter estimates (SE in parentheses) for MDMC and FN-MDMC. Models were fitted to the data of individual participants for the visual-tactile (V-T, Experiment 1) and visual-auditory (V-A, Experiment 2) tasks (each N = 30).

Task	Model	μ_R	μ_{RN}	σ_R	α	b	μ_C	A_V	τ_V	$A_{T/A}$	$\tau_{T/A}$
V-T	MDMC	299 (5.0)	-	31 (1.5)	3.3 (0.11)	64 (2.5)	0.62 (0.03)	12 (0.7)	39 (1.4)	5.6 (0.3)	27 (0.8)
	FN-MDMC	312 (5.2)	299 (5.4)	38 (1.6)	3.1 (0.05)	68 (2.5)	0.74 (0.04)	18 (0.5)	37 (0.7)	6.8 (0.2)	23 (1.0)
V-A	MDMC	300 (7.3)	-	38 (1.8)	3.0 (0.07)	67 (2.8)	0.70 (0.03)	14 (0.5)	50 (0.8)	6.0 (0.2)	34 (0.8)
	FN-MDMC	305 (7.0)	294 (6.5)	38 (1.2)	3.8 (0.07)	65 (2.0)	0.69 (0.03)	15 (0.5)	45 (1.8)	5.0 (0.2)	30 (1.1)

*Note***.** Unit of measurement for μ_R, μ_{RN}, σ_R is the millisecond (*ms*), while the unit of μ_C is $\frac{1}{ms}$.

References

Morey, R.D. (2008). Confidence intervals from normalized data: A correction to Cousineau (2005). *Tutorials in Quantitative Methods for Psychology* 4(2), 61-64.
